# Translation and validation of European organization for research and treatment of cancer quality of life Questionnaire -C30 into Moroccan version for cancer patients in Morocco

**DOI:** 10.1186/1756-0500-7-228

**Published:** 2014-04-10

**Authors:** Chakib Nejjari, Samira El Fakir, Karima Bendahhou, Karima El Rhazi, Naima Abda, Ahmed Zidouh, Abdelatif Benider, Hassan Errihani, Rachid Bekkali

**Affiliations:** 1 Department of Epidemiology and Public health, Faculty of Medicine, University Sidi Mohammed Ben Abdellah, Fez, Morocco; 2 Fondation Lalla Salma Prevention and Treatment of Cancers, Rabat, Morocco; 3 Oncology Center Ibn Rochd, Casablanca, Morocco; 4 National Institute of Oncology, Rabat, Morocco

**Keywords:** Cancer, Quality of life, Reliability, Validity

## Abstract

**Background:**

Understanding the effects of cancer on the quality of life of affected patients is critical to clinical research as well as to optimal management and care. The aim of this study was to adapt the European Organization for Research and Treatment of Cancer Quality of Life Questionnaire-C30 (EORTC QLQ-C30) questionnaire into Moroccan Arabic and to determine its psychometric properties. After translation, back translation and pretesting of the pre-final version, the translated version was submitted to a committee of professionals composed by oncologists and epidemiologists. The psychometric properties were tested in patients with cancer. Internal consistency was tested using Cronbach’s alpha and the test-retest reliability using interclass correlation coefficients. Construct validity was assessed by examining item-convergent and divergent validity. It was also tested using Spearman’s correlation between QLQ-C30 scales and EQ-5D.

**Results:**

The study was conducted in 125 patients. The Moroccan version was internally reliable, Cronbach’s α was 0.87 for the total scale and ranged from 0.34 to 0.97 for the subscales. The intraclass correlation coefficient of the test-retest reliability ranged from 0.64 for “social functioning” to 0.89 for “physical activities” subscales. The instrument demonstrated a good construct and concomitant validity.

**Conclusions:**

We have developed a semantically equivalent translation with cultural adaptation of EORTC QLQ-C30 questionnaire. The assessment of its measurement properties showed that it is quite reliable and a valid measure of the effect of cancer on the quality of life in Moroccan patients.

## Background

Understanding the effects of cancer on the quality of life of affected patients is critical to clinical research as well as to optimal management and care. Measurement of quality of life is helpful in guiding management decisions. Furthermore, it is useful to monitor the effect of therapy on patients’ quality of life. The use of quality of life measures is usually welcomed by patients who wish to express their concerns.

Patients with cancer, enter therapy with the recognition that therapy aimed to cure is often accompanied by side effects that have a negative impact on their quality of life [1]. In recent years, many clinical cancer treatment research protocols have included a quality of life feature to evaluate the balance between side effects and quality of life during sometimes highly toxic treatment regimens [2,3].

Quality of life addresses not only functional abilities, but also symptoms, side effects, and other issues such as social, psychological, spiritual, family, and financial aspects. The assessment of quality of life is becoming a standard component in the overall care of patients with cancer. Its main use in clinical trials has been to provide an additional outcome measure in the comparisons of different oncological treatment regimens.

In Morocco, there is no experience with patient-reported measurements of health-related quality of life in cancer patients. The availability of valid and reliable Moroccan version of quality of life instruments is critical to collect such measurement. Any tool designed to measure quality of life should be multidimensional, subjective, useful in setting, valid, and reliable.

One of the most popular instruments used with cancer patients is the EORTC QLQ-C30 [4].

Culturally adapting an existing instrument rather than composing a new one has several advantages. The cost and time-consuming process of developing a new test is avoided, and the use of standard measure permits cross-cultural comparisons [5].

Cross-cultural adaptation of a quality of life instrument is a step-wise process [5,6].

This incremental methodological approach has become even more essential as increasing experiences have accumulated about cultural differences in the measurement of quality of life, and the recognition of different type of “equivalence” between cultures [7]. However, most adaptation process consists of two primary steps: assessment of the equivalence between the source instrument and the instrument being adapted, and evaluation of the measurement properties of the adapted instrument.

The aims of the current study were to translate into Moroccan Arabic and culturally adapt the EORTC QLQ-C30 (version 3.0), and to assess the psychometric properties of this translation.

## Methods

### Translation and cultural adaptation of the EORTC QLQ-C30

The EORTC QLQ-C30 is a 30-item questionnaire composed of nine multi-item scales and six single items. We followed the published guidelines for cross-cultural adaptation of health-related quality of life measurements [5,8]. As only well-educated Moroccans have a good command of the official language in Morocco, Standard-Arabic, we translated the questionnaire into the oral language commonly spoken among Moroccan people: Moroccan Arabic. Some words could not be translated literally into Moroccan-Arabic, so they were replaced with a short description. For example, there are no Moroccan-Arabic equivalents for the words ‘Hobby’ or ‘quality’. In these cases a Standard-Arabic word was used in addition to the short description.

### Patient recruitment

Between October and November 2009, cancer patients were recruited from the two main oncology centers in the country (National institute of oncology in Rabat and oncology center of Ibn Rochd hospital in Casablanca). Patients attending these two centers are from around the country (north, center and south). The sample size was defined according to Streiner curve [9] for an ICC of 0.70 and a precision of ± 0.10. Patients were eligible if they were at least 18 years old, had a confirmed diagnosis of cancer and spoke Moroccan Arabic. Ethical approval was obtained from the ethics committees in the University Hospital Center Hassan II in Fez- Morocco and all the subjects were informed of the conditions related to the study and gave their written, informed consent.

### Instruments and procedures

The Arabic version of the EORTC QLQ-C30 were administered to patients by two different interviewers. The order of interviewers was randomly defined.

The same questionnaire was re- administered three to ten days later to assess reproducibility.

All participants were also asked to fill in the Euroquol 5 Dimension (EQ-5D) questionnaire which is a standardized instrument to measure health outcome. It provides a simple descriptive profile and a single index value of health status ranging from 0 (death) to 1 (perfect health). EQ-5D consists of five questions covering the dimensions of mobility, self-care, usual activities, pain/discomfort and anxiety/depression, each with three levels of response. The psychometric properties of the Moroccan version were adequate. The Kappa coefficient ranged from 0.49 to 0.92, the intraclass correlation coefficient (ICC) was 0.92 and good correlations between EQ-5D and SF-36 dimensions were noted (r = 0.53 to 0.85) [10].

Participants provided socio-demographic and clinical data and a measure of pain on a visual analogical scale(VAS).

### Statistical analysis

Scores on the items and scales were linearly transformed to a scale from 0 to 100. A high score for a functional scale represents a healthy level of functioning, a high score for the global health status represents a high quality of life, but a high score for a symptom scale/item represents a high level of symptomatology [11].

Descriptive statistics were generated to evaluate missing data and score distributions (i.e. mean, range, floor and ceiling effects).

Internal consistency reliability of the multi-item scales was assessed by Cronbach’s coefficient α. A value of 0.70 or greater was considered as adequate [12]. Inter-rater and test-retest reliabilities were assessed by the intraclass correlation coefficient (ICC), derived from a two-way analysis of variance in a random effect model.

Multitrait scaling analysis were employed to examine item-convergent validity (item-scale correlations should be >0.40) and item-discriminant validity (items should correlate significantly higher with their own scale than with other scales). Construct validity was also tested using Spearman’s correlation between QLQ-C30 scales and EQ-5D.

All statistical analyses were performed using SPSS 17.0.

## Results

### Sample socio-demographic and clinical characteristics

125 patients participated in the study, 65 were recruited in Rabat and 60 in Casablanca. Sociodemographic and clinical characteristics of the study sample are shown in Table 1. Participants ranged in age from 19 to 81 years; the mean age was 44 years (*SD*: 16). 54% were males and 57% were illiterate. The most common primary cancer diagnoses were cancer of lung (17%), breast (16%) and cavum (9%). 67 patients underwent the third assessment.

**Table 1:** Patients’ sociodemographic and clinical characteristics

	**N (%)**
**Center**	
Rabat	65 (52.0)
Casablanca	60 (48.0)
**Gender**	
Female	57 (45.6)
Male	68 (54.4)
**Marital status**	
Single	26 (20.8)
Married	80 (64.0)
Widowed	6 (4.8)
Divorced	5 (4.0)
**Employment status**	
Employed	33 (27,5)
Unemployed	29 (24.2)
Student	4 (3.3)
House wife	49 (40.8)
Retired	5 (41.7)
**Education**	
Illiterate	71 (57.3)
Primary	19 (15.3)
Secondary	30 (24.2)
University	4 (3.2)
**Residence**	
Urban	80 (64.5)
Rural	44 (35.5)
**Primary cancer diagnosis**	
Lung	21 (16.8)
Breast	20 (16.0)
Cavum	11 (8.8)
Cervical cancer	10 (8.0)
Colorectal	10 (8.0)
Stomach	4 (3.2)
**Age** mean (SD)	44.2 (16.0)
**EQ-5D** mean (SD)	0.49 (0.35)
**Third assessment (test-retest)**	85 (68.0)

### Acceptability

The average time to complete the QLQ-C30 was 10 min. On average, missing data rate for items was 1.1% (range 0–1.6%). The items that had missing responses were item 4 of “Physical functioning” scale, items 6 and 7 of “Role functioning”, item 8 “dyspnoea”, items 18 and 19 of “fatigue” and “pain” and item 30 of “global health status”.

### Descriptive statistics

Scores distributions are shown in Table 2. The scores for different scales ranged from 43.4 to 88.7. “Role functioning” scale had the lower score (median 33.3), “social functioning” scale had a median score at 100. Financial difficulties were common with a median at 100. The most frequent symptoms were nausea and vomiting, appetite loss and pain.

**Table 2:** Subscale scores and floor and ceiling effect

	**N**	**Median**	**Mean**	**Standard deviation**	**Floor effect**^**a **^**(%)**	**Ceiling effect**^**b **^**(%)**
Physical functioning	125	60.00	53.86	31.76	7.9	10.3
Role functioning	125	33.33	43.41	43.9	42.1	30.2
Emotional functioning	124	66.67	64.07	33.00	9.5	27.0
cognitive functioning	125	66.67	68.80	31.11	4.8	38.1
Social functioning	125	100	88.67	24.10	3.2	73.8
Global health status	125	50.00	53.25	30.31	7.1	11.9
Fatigue	125	44.44	45.56	33.15	19.0	11.1
Nausea and vomiting	125	66.67	64.13	35.67	11.1	36.5
Pain	125	66.67	60.27	38.03	16.7	33.3
Dyspnoea	124	0.00	27.15	35.65	57.1	9.5
Insomnia	125	0.00	33.86	42.33	57.9	20.6
Appetite loss	125	66.67	61.33	41.35	24.6	44.4
Constipation	125	0.00	26.93	38.73	62.7	15.1
Diarrhoea	125	0.00	18.40	30.95	68.3	6.3
Financial difficulties	125	100	77.33	36.32	13.5	65.9

^a^percentage of the lowest modality.

^b^percentage of the highest modality.

High ceiling or floor effects were observed for the “Role functioning”, “cognitive functioning” and “social functioning” scales and for most of the single item symptom scales (Table 2).

### Reliability

Cronbach’s α was 0.87 for the total scale and ranged from 0.34 for “cognitive functioning” to 0.94 for “role functioning”, indicating good internal consistency (Table 3).

**Table 3:** Internal consistency and reliability of the EORTC QLQ-C30

	**Cronbach’s α**	**Reliability ICC (IC 95%)**	
		**Inter-ratter**	**Test-retest**
Physical functioning	0.84	0.92 [0.88-0.94]	0.89 [0.85-0.93]
Role functioning	0.94	0.82 [0.75-0.87]	0.80 [0.71-0.87]
Emotional functioning	0.89	0.82 [0.76-0.87]	0.82 [0.74-0.88]
cognitive functioning	0.34	0.83 [0.77-0.88]	0.72 [0.61-0.81]
Social functioning	0.80	0.78 [0.69-0.84]	0.64 [0.50-0.75]
Global health status	0.87	0.84 [0.78-0.89]	0.76 [0.66-0.84]
Fatigue	0.83	0.82 [0.75-0.87]	0.84 [0.76-0.89]
Nausea and vomiting	0.74	0.83 [0.77-0.88]	0.78 [0.67-0.85]
Pain	0.78	0.90 [0.86-0.93]	0.82 [0.74-0.88]
Dyspnoea	-	0.78 [0.68-0.84]	0.78 [0.68-0.85]
Insomnia	-	0.73 [0.63-0.81]	0.74 [0.62-0.82]
Appetite loss	-	0.83 [0.77-0.88]	0.75 [0.65-0.83]
Constipation	-	0.89 [0.85-0.92]	0.82 [0.73-0.88]
Diarrhoea	-	0.78 [0.70-0.84]	0.75 [0.64-0.83]
Financial difficulties	-	0.74 [0.65-0.81]	0.80 [0.71-0.87]

Inter-ratter reliability was substantial for the nine scales and the independent items (Table 3).

Test-retest reliability was assessed using the intraclass correlation coefficient, which ranged from 0.64 for “social functioning” to 0.89 for “physical activities”.

### Construct validity

All items exceeded the 0.4 criterion for convergent validity in all scales (Table 4).

**Table 4:** Multitrait scaling analysis of the EORTC QLQ-C30 questionnaire (Moroccan Arabic Version)

**Scales**	**Item convergent validity (item-own scale correlation)**	**Item convergent validity scaling succes**^**a**^	**Item discriminant validity scaling succes**^**b**^
Physical functioning	0.72-0.86	5/5	40/40
Role functioning	0.98-0.98	2/2	16/16
Emotional functioning	0.85-0.89	4/4	32/32
cognitive functioning	0.73-0.82	2/2	16/16
Social functioning	0.89-0.92	2/2	16/16
Global health status	0.94-0.94	2/2	16/16
Fatigue	0.81-0.89	3/3	20/24
Nausea and vomiting	0.89-0.90	2/2	16/16
Pain	0.90-0.93	2/2	16/16

^a^Number of item-scale correlations greater than 0.40/total number of item-scale correlations.

^b^number of correlations of items with own scales significantly higher than correlations with other scale/total number of correlations.

Item-discriminant validity was 100% successful for all items except item 10, 12 and 18. “Fatigue” items were highly correlated with “Physical functioning” scale and item 10 was highly correlated with “global health status”.

The EQ-5D was highly correlated with “Physical functioning” and “Emotional functioning”, and moderately correlated with “Role functioning”, “Global health status” and “Fatigue”. VAS pain was moderately correlated with “pain” scale (Table 5).

**Table 5:** Correlations between EORTC QLQ-C30 scales, EQ-5D and VAS pain

	**EORTC QLQ-C30**								
	**PF**	**RF**	**EF**	**CF**	**SF**	**GHS**	**FA**	**NV**	**PA**
EQ-5D	0.66	0.50	0.43	0.44	0.22	-0.41	0.55	0.27	0.62
VAS pain	-0.25	-0.32	-0.25	-0.32	-0.06	0.27	-0.28	-0.16	-0.59

## Discussion

In this paper, we report the result of a cross-cultural adaptation and evaluation of the psychometric properties of the Moroccan Arabic version of the EORTC QLQ-C30.

This instrument has been developed in 1987 by the European Organisation for Research and Treatment of Cancer. The EORTC QLQ-C30 has been internationally validated [13-17], and is currently available in more than 81 languages (http://www.eortc.be). It was validated to use among Moroccan ethnic minority cancer patients in the Netherlands [18]. However the majority of Moroccan immigrants living in Netherlands were from rural areas of north Morocco where the most common oral language is “Tarifit” and where the educational and literacy levels are lower than national standards. To our knowledge, it is the first Moroccan Arabic validation of a cancer quality of life instrument. This new validated scale will provide information on the functional health and well-being of Moroccan patients in order to provide them with optimal health care services.

To obtain this scale, we followed the international guidelines for cross-cultural adaptation of health-related quality of life measures [5,8].

The average time required to complete the questionnaire was similar to that reported in earlier studies [4,15,17,18].

Time between test and retest was three days on average. Streiner and Norman indicate that expert opinions regarding the appropriate interval vary from one hour to one year, depending on the task, but generally, a retest interval of 2 to 14 days is usually used [19].

The reliability and validity of the Moroccan Arabic version of the EORTC QLQ-C30 were satisfactory. The hypothesized scale structure of the questionnaire was largely confirmed. Cronbach’s alpha coefficient was high for all scales except “cognitive functioning”, indicating adequate internal reliability. Problems with the psychometric properties of “cognitive functioning” scale have been reported in other studies [4,18]. Inter-ratter and test-retest reliability were confirmed by the ICC for the subscales, ICC inter-ratter ranged from 0.73 to 0.92 and test-retest from 0.64 to 0.89.

Construct validity was assessed by testing convergent and discriminant validity of the items, and the associations between the scores of each domain and the EQ-5D score. The most common concurrent scale used in validation studies was the SF36. We could not use this scale because it included 36 items, it could be hard for patients to complete the EORTC QLQ-C30 questionnaire twice and the SF36. The EQ-5D which is a short scale was preferred, especially since administration of the questionnaire need more time than auto-administration.

All items exceeded the 0.4 criterion for convergent validity on all scales. Item-discriminant validity was 100% successful for all items except three. “Fatigue” items were highly correlated with “physical functioning” scale, this result was also observed in the validation study among the Moroccan group living in Netherlands [18].

VAS pain was not highly correlated with “pain” scale because the EORTC QLQ-C30 pain scale measures pain during the past week and VAS pain gives an instantaneous measure during the interview.

Known group’s validity was not tested because the number of missing data about disease stage and treatment status was high.

Because of the high level of illiteracy, an interviewer had administered the questionnaire for all patients. Unlike northern countries, the questionnaire could not be used as an auto-administered questionnaire except for a minority of Moroccan population.

One of the limitations of this study is that responsiveness over time is not performed. We would recommend that additional studies be carried out with patients under active treatment in order to document the responsiveness.

Despite the fact that Arabic language is commonly spoken across the country, there are some other regional languages such as “Tarifit”, “Tamazight” and “Tachelhit”. But, the majority of these people speak also Arabic. Further validation should be specifically performed in these regions because inclusion of these patient groups in local or national clinical studies is essential.

## Conclusion

We conclude that the Moroccan Arabic version of the EORTC QLQ-C30 is a reliable and valid measure of the quality of life in cancer patients and can be used in cohort studies. However, confirmation of its responsiveness requires a formal study assessing the effect of a specific therapeutic intervention.

## Abbreviations

EORTC QLQ-C30: the European Organization for Research and Treatment of Cancer Quality of Life Questionnaires-C30; EQ-5D: Euroquol 5 Dimension; ICC: Intraclass correlation coefficient; SF-36: 36-Item Short Form Health Survey; VAS: Visual analogical scale; SD: Standard deviation.

## Competing interests

The author(s) declare that they have no competing interests.

## Authors’ contributions

CN has contributed to conception and design, acquisition of data, analysis and interpretation of data, have been involved in revising the manuscript critically and have given final approval of the version to be published; SE has contributed to conception and design, interpretation of data and have been involved in drafting the manuscript; KB has made substantial contribution to conception and design of data and has been involved in drafting the manuscript; KE has been involved in drafting the manuscript and have given final approval of the version to be published. NA has contributed to conception and design of data; AZ has contributed to conception and design of data. AB has made substantial contributions to conception and design of data; HE has made substantial contributions to conception and design of data; RB has reviewed the manuscript and has given final approval of the version to be published. All authors read and approved the manuscript.

## References

[B1] Redd WH Montgomery GH DuHamel KN Behavioral intervention for cancer treatment side effects J Natl Cancer Inst 2001 93 11 810 823 10.1093/jnci/93.11.810 11390531

[B2] Gelber RD Goldhirsch A Cavalli F Quality of life adjusted evaluation of adjuvant therapies for operable breast cancer Ann Intern Med 1991 114 8 621 628 10.7326/0003-4819-114-8-621 2003707

[B3] Johnson J Temple R Food and drug administration requirements for approval of new anticancer drugs Cancer Treat Rep 1985 69 1155 1159 4042094

[B4] Aaronson NK Ahmedzai S Bergman B The European Organisation for Research and treatment of Cancer QLQ-C30: a quaity of life instrument for use in internetional clinical trials in oncology J Nat Cancer inst 1993 85 364 376 10.1093/jnci/85.5.365 8433390

[B5] Guillemin F Bombardier C Beaton D Cross-cultural adaptation of health-related quality of life measures: literature review and proposed guidelines J Clin Epidemiol 1993 46 12 1417 1432 10.1016/0895-4356(93)90142-N 8263569

[B6] Lohr KN Aaronson NK Alomso J Burnam MA Patrick DL Perrin EB Roberts JS Evaluating quality-of-life and health-status instrument: development of scientific review criteria Clin Ther 1996 18 979 992 10.1016/S0149-2918(96)80054-3 8930436

[B7] Herdman M Fox-Rushby J Badia X A model of equivalence in the cultural adptation of HRQL instruments: the universalist approach Qual Life Res 1998 7 323 335 10.1023/A:1008846618880 9610216

[B8] Beaton D Bombardier C Guillemin F Ferraz MB Guidelines for the process of cross-cultural adaptation of self-report measures SPINE 2000 25 24 3186 3191 10.1097/00007632-200012150-00014 11124735

[B9] Streiner DL Norman GR Health Measurment Scales. Apractical Guide to Their Development and use 2003 Third Oxford: Oxford University Press

[B10] Khoudri36th Congress of the SRLF 2008 Paris:

[B11] Fayers PM Aaronson NK Bjordal K Groenvold M Curran D Bottomley A The EORTC QLQ-C30 Scoring Manual 2001 3 Brussels: European Organisation for Research and Treatment of Cancer

[B12] Cronbach LJ Coefficient alpha and the internal structure of tests Psychometrika 1951 16 297 334 10.1007/BF02310555

[B13] Aaronson NK Bullinger M Ahmedzai S A modular approach to quality-of-life assessment in cancer clinical trials Recent Results Cancer Res 1988 111 231 249 10.1007/978-3-642-83419-6_27 2459750

[B14] Aaronson NK Ahmedzai S Bergman B Bullinger M Cull A Duez NJ Filiberti A Flechtner H Fleishman SB de Haes JCJM Kaasa S Klee M Osoba D Razavi D Rofe PB Schraub S Sneeuw K Sullivan M Takeda F The European Organization of Research and Treatment of cancer QLQ-C30: a quality-of-life instrument for use in internationl clinical trials in oncology J Nat Cancer Inst 1993 85 5 365 376 10.1093/jnci/85.5.365 8433390

[B15] Montazeri A Harirchi I Vahdani M Khaleghi F Jarvandi S Ebrahimi M The European Organization for Research and Treatment of Cancer Qualitu of Life Questionnaire (EORTC QLQ-C30): translation and validation study of the Iranian version Support Care Cancer 1999 7 6 400 406 10.1007/s005200050300 10541982

[B16] Yun YH Park YS Lee ES Bang SM Heo DS Park SY You CH West K Validation of the Korean version of the EORTC QLQ-C30 Qual Life Res 2004 13 4 863 868 15129896 10.1023/B:QURE.0000021692.81214.70

[B17] Zhao H Kanda K Translation and validation of the standard Chinese version of the EORTC QLQ-C30 Qual Life Res 2000 9 2 129 137 10.1023/A:1008981520920 10983477

[B18] Hoopman R Muller MJ Terwee CB Aaronson NK Translation and validation of the EORTC QLQ-C30 for use among Turkish and Moroccan ethnic minority cancer patients in the Netherlands Eur J Cancer 2006 42 1839 1847 10.1016/j.ejca.2005.08.047 16750911

[B19] Guyatt GH Feeny DH Patrick DL Measurment health-related quality of life Ann Intern Med 1993 118 622 629 10.7326/0003-4819-118-8-199304150-00009 8452328

